# Nonalcoholic Fatty Liver Disease, Liver Fibrosis and Cardiovascular Disease in the Adult US Population

**DOI:** 10.3389/fendo.2021.711484

**Published:** 2021-07-26

**Authors:** Stefano Ciardullo, Rosa Cannistraci, Simone Mazzetti, Andrea Mortara, Gianluca Perseghin

**Affiliations:** ^1^ Department of Medicine and Rehabilitation, Policlinico di Monza, Monza, Italy; ^2^ Department of Medicine and Surgery, Università degli Studi di Milano Bicocca, Milan, Italy; ^3^ Clinical Cardiology, Policlinico di Monza, Monza, Italy

**Keywords:** NAFLD, MAFLD, fibrosis, CVD, fibroscan and transient elastography

## Abstract

**Background:**

Cardiovascular disease (CVD) risk is higher in patients with nonalcoholic fatty liver disease (NAFLD).

**Aim:**

To evaluate whether this can be attributed to the link between NAFLD and known CVD risk factors or to an independent contribution of liver steatosis and fibrosis.

**Methods:**

This is an analysis of data from the 2017-2018 cycle of the National Health and Nutrition Examination Survey. We included participants older than 40 years with available data on vibration-controlled transient elastography (VCTE) and without viral hepatitis and significant alcohol consumption. Steatosis and fibrosis were diagnosed by the median value of controlled attenuation parameter (CAP) and liver stiffness measurement (LSM), respectively. History of CVD was self-reported and defined as a composite of coronary artery disease and stroke/transient ischemic attacks.

**Results:**

Among the 2734 included participants, prevalence of NAFLD was 48.6% (95% CI 45.1-51.4), 316 participants (9.7%, 95% CI 8.1-11.6) had evidence of significant liver fibrosis and 371 (11.5%, 95% CI 9.5-13.9) had a history of CVD. In univariate analysis, patients with CVD had a higher prevalence of steatosis (59.6% *vs* 47.1%, p=0.013), but not fibrosis (12.9% *vs* 9.3%, p=0.123). After adjustment for potential confounders in a multivariable logistic regression model, neither steatosis nor significant fibrosis were independently associated with CVD and heart failure.

**Conclusions:**

In this population-based study, we did not identify an independent association between steatosis and fibrosis and CVD. Large prospective cohort studies are needed to provide a more definitive evidence on this topic.

## Introduction

As a consequence of the increasing rates of obesity worldwide, nonalcoholic fatty liver disease (NAFLD) has become a public health problem (1). It is estimated that it affects a quarter of the adult population (2) and its prevalence is expected to grow in the near future, given its high prevalence in children and adolescents (3). The term NAFLD encompasses a wide range of histologic changes, spanning from simple hepatic steatosis, to leukocyte infiltration and hepatocyte ballooning (nonalcoholic steatohepatitis, NASH) to advanced liver fibrosis and cirrhosis (4).

NAFLD is tightly linked to insulin resistance and the cardiometabolic risk factors encapsulated by the metabolic syndrome, including central obesity, hypertension (5), type 2 diabetes (T2D) (6) and dyslipidemia (7). It is therefore not surprising that patients with NAFLD have a higher cardiovascular disease (CVD) risk, and that CVD represents the most common cause of morbidity and mortality in these patients (8). Nevertheless, it is still uncertain whether this is due to its strict association with known CVD risk factors or to an independent contribution of liver fat (9). Moreover, recent studies identified a relationship between liver fibrosis, which is the strongest predictor of future liver-related events in patients with NAFLD, and CVD risk factors (10).

While the gold-standard technique for staging fibrosis is still liver biopsy (an invasive technique not well suited for screening purposes), several non-invasive imaging methods have been developed (11). Among them, vibration-controlled transient elastography (VCTE) demonstrated good accuracy in identifying subjects with liver steatosis and significant fibrosis (≥F2) and was used in several population-based studies to estimate disease prevalence (12).

Since in the 2017-2018 cycle of the National Health and Nutritional Examination Survey (NHANES) VCTE was performed for the first time in a US nationally representative sample, data from the survey were used in the present study to obtain evidence on the association between liver steatosis and fibrosis and CVD.

## Methods

The present study analyzed data from the 2017-2018 cycle of NHANES. NHANES is a cross-sectional survey conducted in the United States by the National Center for Health Statistics of the Centers for Disease Control and Prevention. It employs a stratified, multistage, clustered probability sampling design to include individuals representative of the general, non-institutionalized population. The complex survey design aims at obtaining enough data on minorities through oversampling of non-Hispanic black, Hispanic and Asian persons, people with low income and older adults. The survey is divided in two parts: a structured interview conducted in the participants home and the mobile examination center (MEC) visit, in which NHANES staff perform a standardized physical examination as well as laboratory tests. Full methodology of data collection is available elsewhere (13). The survey was approved by the Centers for Disease Control and Prevention Research Ethics Review Board and written informed consent was obtained from all adult participants. The present analysis was deemed exempt by the Institutional Review Board at our institution, as the dataset used in the analysis was completely de-identified.

### Clinical and Laboratory Data

Body measurements including height (cm), weight (kg) and waist circumference (cm) were obtained by NHANES staff during the MEC visit; body mass index (BMI) was then calculated as weight in kilograms divided by height in meters squared and obesity defined as a BMI≥30 Kg/m^2^. Participants were considered to have diabetes if they self-reported a previous diabetes diagnosis or the use of anti-diabetic drugs, or had a Hemoglobin A1c (HbA1c) level ≥ 6.5% (48 mmol/mol) during the survey (14).

Laboratory methods for measurements of HbA1c, glucose, total cholesterol, high density lipoprotein (HDL) cholesterol, alanine aminotransferase (ALT), aspartate aminotransferase (AST), γ-glutamyltranspeptidase (GGT), platelet count and albumin are reported in detail elsewhere (15). Current Hepatitis C virus infection was indicated by presence of viral RNA and/or a confirmed antibody test and hepatitis B virus infection as a positive surface antigen test, as described (15). Estimated glomerular filtration rate (eGFR) was computed according to the Chronic Kidney Disease Epidemiology Collaboration (CKD-EPI) equation (16) and CKD was defined as an eGFR < 60 ml/min/1.73 m^2^. Alcohol consumption was estimated based on self-reported data on the amount and frequency of alcohol use within the previous year. The amount of alcohol consumed was reported in standard drinks and converted to grams using a multiplication factor of 14. It was considered significant if >30 g/day for men and >20 g/day for women (17).

Drug use was determined from participant self-report during the in-home questionnaire. Moreover, trained interviewers reviewed participants’ pill bottles for prescription and nonprescription medications and supplements reported to have been taken in the previous month. During the in-home part of the survey, trained interviewers obtained information on self- and proxy-reported health conditions and medical history using the Computer-Assisted Personal Interview (CAPI) system. Among the studied conditions, several questions dealt with previous coronary artery disease (CAD), stroke and transient ischemic attack (TIA) and congestive heart failure (HF). These represent the outcome of interest in the present study. CVD was defined as a previous history of CAD and/or stroke/TIAs. We additionally considered a composite endpoint comprising both CVD and HF (referred here as composite).

### VCTE

In the 2017-2018 cycle, the FibroScan^®^ model 502 V2 Touch (Echosens, Paris, France) equipped with a medium (M) and extra-large (XL) probes was used to perform VCTE. VCTE is a non-invasive technique able to simultaneously evaluate liver steatosis (through the controlled attenuation parameter CAP) and fibrosis (through liver stiffness measurement (LSM)) (11). The exam was performed by NHANES technicians after a 2-day training program with an expert technician. The M probe was used initially unless the machine indicated use of the XL probe. Inter-rater reliability between health technicians and expert FibroScan^®^ technicians was tested on 32 subjects and was reported to be 0.86 for stiffness (mean difference 0.44 ± 1.3 KPa) and 0.94 for CAP (mean difference 4.5 ± 19.8 db/m). Participants were instructed to fast for at least three hours before the exam and were placed in a supine position with the right arm fully abducted and measurements were performed by scanning the right liver lobe through an intercostal approach. CAP and LSM values were expressed in decibels per meter (dB/m) and kilopascals (kPa), respectively. For the present analysis, exams were considered reliable only if at least 10 LSM values were obtained after a fasting time of at least 3 hours, with an interquartile (IQRe) range/median < 30%. Participants were considered to have steatosis (≥S1) if CAP ≥ 274 dB/m, in accordance with a recent landmark study by Eddowes et al. (18). A median LSM ≥8 KPa was considered indicative of significant (≥F2) fibrosis (19).

### Analysis Sample

**Figure 1:**
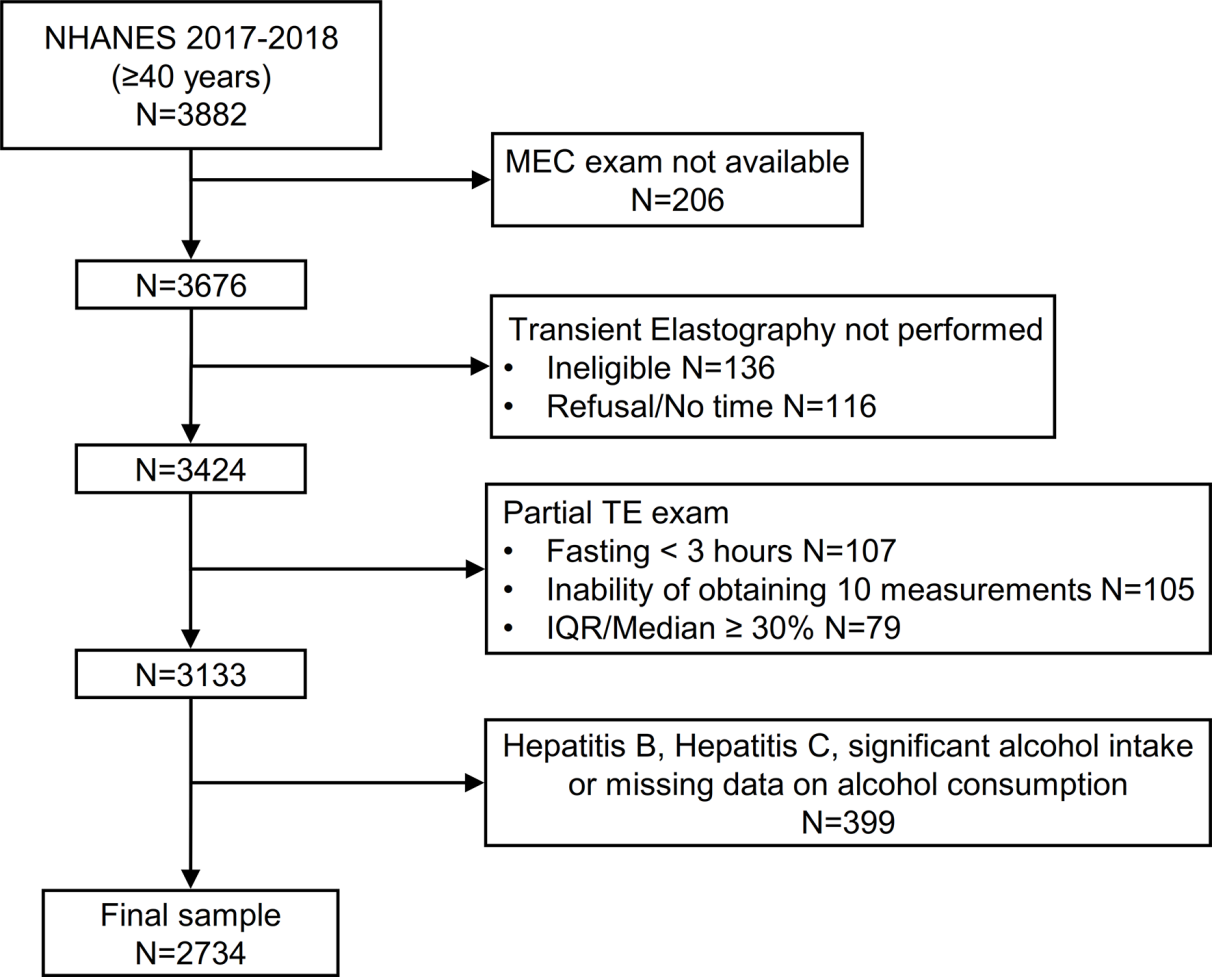
Flow-chart of the study participants. NHANES, National Health and Nutrition Examination Survey.

3676 participants aged ≥40 years attended a MEC visit. We initially excluded 136 participants that were considered ineligible for VCTE for different reasons (unable to lie down, currently pregnant, presence of an implanted electronic medical device, presence of lesions where measurements would be taken) and 116 additional participants without a VCTE exam because of refusal or insufficient time for the examination. Of the remaining 3424 patients, 291 (8.5%) had an incomplete VCTE exam because of fasting for less than 3 hours (n=107), inability of obtaining 10 valid measures (n=105) and an IQR/median LSM value ≥ 30% (n=79). Finally, we excluded patients with evidence of hepatitis B, hepatitis C and significant alcohol consumption (or unavailable data on alcohol intake, n=399) leading to a final sample of 2734 patients (**Figure 1**).

### Statistical Analysis

All analyses were conducted using Stata version 13.0 (StataCorp, College Station, TX), accounting for the complex survey design of NHANES. We used appropriate weighting for each analysis, as suggested by the NCHS. Data are expressed as numbers and weighted proportions for categorical variables and as weighted means ± Standard Error (SE) for continuous variables.

Participants’ characteristics according to the presence or absence of liver steatosis and fibrosis and CVD were compared using linear regression for continuous variables and the design-adjusted Rao-Scott chi-square test for categorical variables. Multivariable logistic regression analysis was performed in order to evaluate the effect of steatosis and fibrosis on different cardiovascular outcomes. A biological plausibility approach was followed for the choice of covariates including known CVD risk factors such as age, sex, race/ethnicity, BMI, diabetes, CKD and cigarette smoke. Multicollinearity was tested by calculating the variance inflation factor (VIF). We found no significant collinearity (VIF ≥ 2) between the variables included in our model. A two-tailed value of p < 0.05 was considered statistically significant.

## Results

Of the 2734 included participants, 1907 (69.8%) were evaluated with the M probe and 827 (30.2%) with the XL probe. In total, 1373 had evidence of NAFLD (weighted prevalence 48.6%, 95% CI 45.7-51.4), with higher rates in men (54.2%, 95% CI 50.2-58.2) than women (43.6, 95% CI 40.0-47.3). Clinical features of patients with and without NAFLD are shown in **Table 1**.

**Table 1:** Features of the study population according to the presence of liver steatosis assessed by the controlled attenuation parameter (CAP).

	Entire cohort (n=2734)	CAP < 274 dB/m (n=1361)	CAP ≥ 274 dB/m (n=1373)	p-value
Age (years)	58.7 ± 0.4	58.3 ± 0.5	59.4 ± 0.6	0.039
Male sex (%)	46.8 ± 0.9	41.7 ± 1.3	52.3 ± 1.8	<0.001
Race-Ethnicity (%)				
Non-Hispanic white	67.2 ± 2.8	68.1 ± 3.2	66.1 ± 2.9	0.093
Hispanic	13.2 ± 1.7	11.5 ± 1.7	15.0 ± 2.1	
Non-Hispanic black	9.9 ± 1.6	11.0 ± 1.8	8.7 ± 1.4	
Non-Hispanic Asian	5.3 ± 0.9	5.4 ± 0.9	5.2 ± 1.0	
Other	4.5 ± 0.8	4.0 ± 1.1	5.0 ± 0.8	
Cigarette smoke (%)				0.252
Never	57.9 ± 1.8	58.4 ± 2.7	57.3 ± 2.1	
Past	29.9 ± 1.2	28.1 ± 1.8	31.8 ± 2.2	
Current	12.3 ± 1.1	13.5 ± 1.6	10.9 ± 1.0	
BMI (Kg/m^2^)	29.8 ± 0.3	27.1 ± 0.2	33.0 ± 0.4	<0.001
**Laboratory parameters**				
HbA1c (%)	5.9 ± 0.0	5.6 ± 0.0	6.2 ± 0.0	<0.001
AST (IU/L)	20.6 ± 0.2	20.5 ± 0.2	21.4 ± 0.3	0.014
ALT (IU/L)	21.9 ± 0.2	19.1 ± 0.3	24.0 ± 0.4	<0.001
GGT (IU/L)	30.7 ± 0.8	24.9 ± 1.1	33.0 ± 1.0	<0.001
Albumin (g/dL)	4.0 ± 0.0	4.1 ± 0.0	4.0 ± 0.0	0.554
Platelet count (109/L)	240.0 ± 3.4	237.6 ± 4.5	242.8 ± 3.0	0.163
Total Cholesterol (mg/dL)	195.7 ± 1.8	199.4 ± 1.8	192.2 ± 2.7	0.011
Triglycerides (mg/dL)	150.7 ± 3.6	124.3 ± 3.0	180.5 ± 6.4	<0.001
HDL-Cholesterol (mg/dL)	54.4 ± 0.5	58.1 ± 0.7	49.5 ± 0.5	<0.001
**Comorbidities**				
Type 2 diabetes (%)	19.3 ± 0.9	9.6 ± 1.0	29.6 ± 1.8	<0.001
Blood pressure category (%)				<0.001
Optimal	25.2 ± 2.1	34.4 ± 2.7	15.4 ± 2.0	
Normal	13.7 ± 1.1	14.5 ± 1.4	12.8 ± 1.4	
High-normal	9.0 ± 1.1	8.0 ± 1.5	10.0 ± 1.1	
Hypertension	52.2 ± 2.4	43.0 ± 2.7	61.9 ± 2.9	
CKD (%)	10.5 ± 0.9	10.0 ± 1.2	11.0 ± 1.2	0.512
HF (%)	2.2 ± 0.4	1.0 ± 0.2	3.5 ± 0.8	<0.001
CAD (%)	8.5 ± 0.9	6.4 ± 0.8	10.9 ± 1.6	0.013
Stroke (%)	4.2 ± 0.5	3.5 ± 0.7	4.9 ± 0.9	0.265
CVD (%)	11.5 ± 1.0	9.0 ± 0.8	14.1 ± 1.8	0.013
Composite (%)	12.1 ± 1.0	9.4 ± 0.8	15.0 ± 1.9	0.008
Statin use (%)	26.8 ± 1.8	21.1 ± 1.5	32.8 ± 2.5	<0.001

Data are expressed as weighted proportions [± Standard Error (SE)] for categorical variables and as weighted means ± SE for continuous variables. Linear regression and Rao-Scott chi-square test were used to compare groups.

BMI, Body Mass Index; HbA1c, Hemoglobin A1c; AST, aspartate aminotransferase; ALT, alanine aminotransferase; GGT, gamma-glutamyltranspeptidase; HDL, high density lipoprotein; CVD, cardiovascular disease; HF, heart failure; CKD, chronic kidney disease; CAD, coronary artery disease.

Briefly, participants with NAFLD were older, had a higher BMI and were more frequently male, while no significant differences were found in race-ethnicity. Patients with NAFLD showed a less favorable metabolic profile, characterized by lower HDL-C, higher triglycerides, higher HbA1c and liver enzymes. They also showed a higher prevalence of type 2 diabetes and hypertension and were more frequently treated with statins. They showed higher prevalence of most cardiovascular events except for stroke. No significant difference was found in CKD.

**Table 2** shows the clinical features of participants recruited in this analysis, stratified by liver fibrosis. Significant fibrosis was present in 316 patients (weighted prevalence 9.7%, 95% CI 8.1-11.6%), with higher rates in men (12.0%, 95% CI 9.2-15.7) than women (7.6%, 95% CI 6.0-9.5), while an LSM≥10 kPa was present in 183 participants (5.8%, 95% CI 4.6-7.3). Patients with significant fibrosis were more commonly male, had a higher BMI and liver enzymes and no significant difference in age and ethnicity distribution. They showed a higher prevalence of type 2 diabetes, hypertension, CKD and HF, but no difference in CVD, CAD and stroke/TIA.

**Table 2:** Features of the study population according to the presence of significant liver fibrosis assessed by the median liver stiffness measurement (LSM).

	LSM < 8 kPa (n=2418)	LSM ≥ 8 kPa (n=316)	p-value
Age (years)	58.7 ± 0.4	60.6 ± 1.4	0.384
Male sex (%)	45.6 ± 0.9	58.3 ± 4.5	0.015
Race-Ethnicity (%)			0.092
Non-Hispanic white	67.7 ± 2.7	61.8 ± 4.5	
Hispanic	12.7 ± 1.7	17.9 ± 3.0	
Non-Hispanic black	9.7 ± 1.5	11.6 ± 2.8	
Non-Hispanic Asian	5.5 ± 0.9	3.6 ± 1.4	
Other	4.4 ± 0.8	5.0 ± 1.8	
Cigarette smoke (%)			0.083
Never	58.0 ± 1.9	56.3 ± 4.1	
Past	29.2 ± 1.3	35.7 ± 4.0	
Current	12.7 ± 1.1	8.0 ± 1.8	
BMI (Kg/m2)	29.3 ± 0.3	36.3 ± 0.6	<0.001
**Laboratory parameters**			
HbA1c (%)	5.8 ± 0.0	6.5 ± 0.1	<0.001
AST (IU/L)	20.4 ± 0.2	25.3 ± 1.2	0.001
ALT (IU/L)	20.8 ± 0.3	28.3 ± 1.1	<0.001
GGT (IU/L)	26.9 ± 0.7	47.8 ± 3.6	<0.001
Albumin (g/dL)	4.1 ± 0.0	4.0 ± 0.0	0.005
Platelet count (109/L)	241.3 ± 3.9	229.4 ± 3.8	0.069
Total Cholesterol (mg/dL)	197.0 ± 1.9	185.7 ± 4.0	0.004
Triglycerides (mg/dL)	147.5 ± 3.5	193.6 ± 20.3	0.042
HDL-Cholesterol (mg/dL)	54.5 ± 0.5	48.5 ± 1.4	<0.001
**Comorbidities**			
Type 2 diabetes (%)	16.4 ± 0.7	45.9 ± 4.9	<0.001
Blood Pressure category (%)			0.016
Optimal	26.7 ± 2.2	11.4 ± 1.9	
Normal	14.2 ± 1.2	9.0 ± 2.3	
High-normal	8.6 ± 1.0	12.7 ± 5.3	
Hypertension	50.6 ± 2.4	66.8 ± 5.2	
CKD (%)	10.0 ± 0.9	15.2 ± 2.9	0.037
HF (%)	1.9 ± 0.5	5.4 ± 1.3	0.007
CAD (%)	8.2 ± 1.1	11.8 ± 2.3	0.191
Stroke (%)	4.0 ± 0.6	5.5 ± 1.6	0.326
CVD (%)	11.1 ± 1.2	15.4 ± 2.1	0.123
Composite (%)	11.5 ± 1.2	17.7 ± 2.5	0.040
Statin use (%)	26.2 ± 1.7	32.4 ± 5.2	0.196

Data are expressed as weighted proportions [± Standard Error (SE)] for categorical variables and as weighted means ± SE for continuous variables. Linear regression and Rao-Scott chi-square test were used to compare groups.

BMI, Body Mass Index; HbA1c, Hemoglobin A1c; AST, aspartate aminotransferase; ALT, alanine aminotransferase; GGT, gamma-glutamyltranspeptidase; HDL, high density lipoprotein; CVD, cardiovascular disease; HF, heart failure; CKD, chronic kidney disease; CAD, coronary artery disease.

Finally, population features according to CVD status are shown in **Table 3**. CVD was present in 371 participants (weighted prevalence 11.5%, 95% CI 9.5-13.9%). In particular, 8.5% (95% CI 6.8-10.7) had CAD and 4.2% (95% CI 3.2-5.5) had stroke/TIA, while the prevalence of HF was 2.2% (95% CI 1.5-3.3). Patients with CVD were older, more frequently non-Hispanic white and had a higher proportion of current or past smokers. Prevalence of type 2 diabetes, CKD and hypertension was higher in this group, as well as the proportion of patients treated with statins. Patients with CVD showed lower albumin levels and platelet count, but no differences in liver enzymes.

**Table 3:** Features of the study population according to the presence of cardiovascular disease (CVD).

	CVD - (n=2363)	CVD + (n=371)	p-value
Age (years)	57.8 ± 0.4	66.7 ± 0.7	<0.001
Male sex (%)	45.3 ± 1.0	58.3 ± 4.1	0.010
Race-Ethnicity (%)			0.006
Non-Hispanic white	66.2 ± 2.9	74.3 ± 3.5	
Hispanic	13.9 ± 1.9	7.7 ± 1.5	
Non-Hispanic black	9.9 ± 1.6	9.7 ± 1.9	
Non-Hispanic Asian	5.7 ± 1.0	2.0 ± 0.6	
Other	4.3 ± 0.7	6.3 ± 2.2	
Cigarette smoke (%)			<0.001
Never	60.0 ± 1.8	41.5 ± 4.2	
Past	28.4 ± 1.2	41.4 ± 3.3	
Current	11.6 ± 1.1	17.1 ± 2.9	
BMI (Kg/m2)	30.0 ± 0.3	30.1 ± 0.4	0.601
**Laboratory parameters**			
HbA1c (%)	5.8 ± 0.0	6.2 ± 0.1	<0.001
AST (IU/L)	20.9 ± 0.2	21.4 ± 0.7	0.443
ALT (IU/L)	21.6 ± 0.3	20.5 ± 1.0	0.282
GGT (IU/L)	28.2 ± 0.7	34.1 ± 3.3	0.080
Albumin (g/dL)	4.1 ± 0.0	4.0 ± 0.0	<0.001
Platelet count (109/L)	242.7 ± 3.6	221.1 ± 4.2	<0.001
Total Cholesterol (mg/dL)	198.4 ± 2.0	176.6 ± 5.2	0.001
Triglycerides (mg/dL)	150.3 ± 3.7	164.1 ± 7.7	0.058
HDL-Cholesterol (mg/dL)	54.2 ± 0.5	51.2 ± 1.8	0.148
**Comorbidities**			
Type 2 diabetes (%)	16.7 ± 0.9	39.3 ± 3.1	<0.001
Blood pressure category (%)			0.001
Optimal	26.8 ± 2.3	12.6 ± 3.1	
Normal	14.3 ± 1.0	9.3 ± 2.2	
High-normal	9.2 ± 1.3	7.1 ± 1.7	
Hypertension	49.7 ± 2.3	71.0 ± 5.3	
CKD (%)	8.6 ± 0.8	25.2 ± 2.9	<0.001
Statin use (%)	21.7 ± 1.7	65.9 ± 3.6	<0.001
CAP ≥ 274 dB/m	47.1 ± 1.5	59.6 ± 3.8	0.013
LSM ≥ 8 kPa	9.3 ± 0.8	12.9 ± 2.4	0.123

Data are expressed as weighted proportions [± Standard Error (SE)] for categorical variables and as weighted means ± SE for continuous variables. Linear regression and Rao-Scott chi-square test were used to compare groups.

BMI, Body Mass Index; HbA1c, Hemoglobin A1c; AST, aspartate aminotransferase; ALT, alanine aminotransferase; GGT, gamma-glutamyltranspeptidase; HDL, high density lipoprotein; CKD, chronic kidney disease.

### Association Between Liver Steatosis and Fibrosis and Cardiovascular Outcomes

To further examine the relationship between liver disease and CVD, logistic regression analysis was performed, with inclusion of several potential confounders.

**Figure 2:**
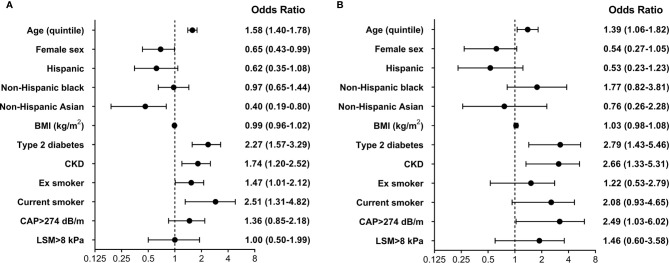
Multivariable logistic regression model evaluating the effect of several predictors on the presence of **(A)** cardiovascular disease (CVD) and **(B)** heart failure (HF). Reference categories for cigarette smoke were never smoker, while the reference category for race-ethnicity was non-Hispanic white. BMI, body mass index; CAP, controlled attenuation parameter; LSM, liver stiffness measurement; CKD, chronic kidney disease.

As shown in **Figure 2**, higher age, presence of type 2 diabetes, past and current cigarette smoke and CKD were associated with higher odds of CVD, while female participants and non-Hispanic Asian ethnicity were identified as protective features. No significant association was found for liver steatosis and fibrosis. Results were confirmed in analyses that considered CAD and cerebrovascular disease as separate outcomes. The same predictors were included in a model investigating HF. In this analysis higher age, presence of type 2 diabetes, CKD, current cigarette smoke and liver steatosis were associated with increased odds, while no independent contribution was identified for BMI, race-ethnicity and liver fibrosis. Lack of a significant association between measures of steatosis and fibrosis and CVD outcomes was confirmed when CAP and LSM were included as continuous variables in multivariable models.

## Discussion

In the present population-based cross-sectional study including 2734 US adults, we show that patients with NAFLD have a worse cardio-metabolic risk profile and a higher prevalence of CVD. Nonetheless, the association between VCTE-diagnosed NAFLD and liver fibrosis and CVD was not significant after adjustment for known cardiovascular risk factors.

The topic of a potential independent contribution of NAFLD and liver fibrosis to the occurrence of cardiovascular events is still debated in the literature, in large part due to the difficulties related to adjustment for CVD risk factors that tend to cluster in patients with NAFLD and to accurately identify patients with fibrosis in unselected populations (20). In particular, a large meta-analysis including 16 observational cohort studies with > 34.000 individuals showed that NAFLD (diagnosed by either liver biopsy or imaging methods, consisting mostly in liver ultrasound) was associated with an OR of 1.64 for fatal and non-fatal CVD events (21). This association remained significant even when only studies adjusting for several known risk factors were considered. On the other hand, a recent large cohort study including >120.000 patients with NAFLD and >9.000.000 controls performed using electronic primary care records from four European countries showed that a diagnosis of NAFLD was not associated with incident myocardial infarction and stroke after adjustment for cardiovascular risk factors (9).

Fewer studies evaluated the impact of liver fibrosis on CVD outcomes and reached different conclusions. In a cohort study including 285 patients with available liver biopsy followed for a median of 5.2 years, presence of stage 3-4 fibrosis was independently associated with an increased risk of CVD, even though only 26 events occurred (22). Conversely, Hagstrom et al. followed 603 patients with biopsy-proven NAFLD and reported that no histological parameter, including presence of NASH and fibrosis stage, was independently associated with CVD after adjustment for age, sex, type 2 diabetes, smoking and triglycerides levels (23). Our study expands these results by focusing on the general population (thereby providing estimates with a high degree of external validity), rather than on patients with known NAFLD followed at tertiary care centers and employing VCTE as a non-invasive method to estimate the degree of liver fibrosis.

The present study has several limitations that deserve to be acknowledged. First, its cross-sectional nature does not allow to evaluate temporal trends and therefore prove the existence of cause-effect relationships. Moreover, the design does not allow to evaluate the association between NAFLD and fatal CVD outcomes. Further prospective cohort studies with VCTE in the general population are still needed. Second, diagnosis of CVD and HF was based exclusively on participants self-report and recall bias might be present. Third, even though VCTE has been widely validated as an accurate measure of liver fibrosis, it cannot be considered a gold standard technique, as its performance is reduced in severely obese patients and in the setting of ascites, acute inflammation and congestion (24). It is therefore possible that the association found between CAP and HF in the current study is at least in part attributable to this technological limitation. On this topic, a previous study including 10 patients with decompensated HF showed that liver stiffness decreased significantly upon recompensation in all of them, following a median weight loss of 3.0 kg (25). Similar results were obtained in a second study of 27 hospitalized patients with HF, in which, during hospitalization, liver stiffness decreased in 18 patients (including all patients with baseline measurement above 13 kPa) (26).

It should be stressed, however, that liver biopsy (the gold standard technique) is an invasive procedure, prone to both sampling variability and bleeding episodes and therefore not applicable to a large population-based study (27, 28).

In conclusion, this large population-based study shows that 1) VCTE-diagnosed NAFLD and associated significant fibrosis are common in the general US population and 2) neither steatosis nor fibrosis are associated with cardiovascular comorbidities after accounting for known CVD risk factors. Given that the topic is still debated in the literature and the paucity of data on liver fibrosis and CVD events, large prospective cohort studies are needed to provide more definitive evidence on this topic.

## Data Availability Statement

Publicly available datasets were analyzed in this study. This data can be found here: https://wwwn.cdc.gov/nchs/nhanes/ContinuousNhanes/Default.aspx#:~:text=What%20is%20Continuous%20NHANES%3F,non%2Dinstitutionalized%2C%20U.S.%20population.

## Ethics Statement

Ethical review and approval were not required for the study on human participants in accordance with the local legislation and institutional requirements. The patients/participants provided their written informed consent to participate in this study.

## Author Contributions

SC and GP designed the study, wrote, reviewed, and edited the manuscript. SC researched and analyzed data. RC, SM, and AM reviewed and contributed in editing the manuscript. GP is the guarantor of this work. All authors contributed to the article and approved the submitted version.

## Conflict of Interest

The authors declare that the research was conducted in the absence of any commercial or financial relationships that could be construed as a potential conflict of interest.

## Publisher’s Note

All claims expressed in this article are solely those of the authors and do not necessarily represent those of their affiliated organizations, or those of the publisher, the editors and the reviewers. Any product that may be evaluated in this article, or claim that may be made by its manufacturer, is not guaranteed or endorsed by the publisher.
